# Current achievements and future perspectives in whole-organ bioengineering

**DOI:** 10.1186/s13287-015-0089-y

**Published:** 2015-06-01

**Authors:** Andrea Peloso, Abritee Dhal, Joao P Zambon, Peng Li, Giuseppe Orlando, Anthony Atala, Shay Soker

**Affiliations:** IRCCS Policlinico San Matteo, Department of General Surgery, University of Pavia, Viale Golgi 19, Pavia, 27100 Italy; Wake Forest Institute for Regenerative Medicine, Medical Centre Boulevard, Winston-Salem, NC 27157 USA; Department of General Surgery Affiliated Hospital of Nantong University, Nantong University, Nantong, Jiangsu 226001 China; Wake Forest School of Medicine, Medical Centre Boulevard, Winston-Salem, NC 27517 USA

## Abstract

Irreversible end-stage organ failure represents one of the leading causes of death, and organ transplantation is currently the only curative solution. Donor organ shortage and adverse effects of immunosuppressive regimens are the major limiting factors for this definitive practice. Recent developments in bioengineering and regenerative medicine could provide a solid base for the future creation of implantable, bioengineered organs. Whole-organ detergent-perfusion protocols permit clinicians to gently remove all the cells and at the same time preserve the natural three-dimensional framework of the native organ. Several decellularized organs, including liver, kidney, and pancreas, have been created as a platform for further successful seeding. These scaffolds are composed of organ-specific extracellular matrix that contains growth factors important for cellular growth and function. Macro- and microvascular tree is entirely maintained and can be incorporated in the recipient’s vascular system after the implant. This review will emphasize recent achievements in the whole-organ scaffolds and at the same time underline complications that the scientific community has to resolve before reaching a functional bioengineered organ.

## Introduction

**Fig. 1:**
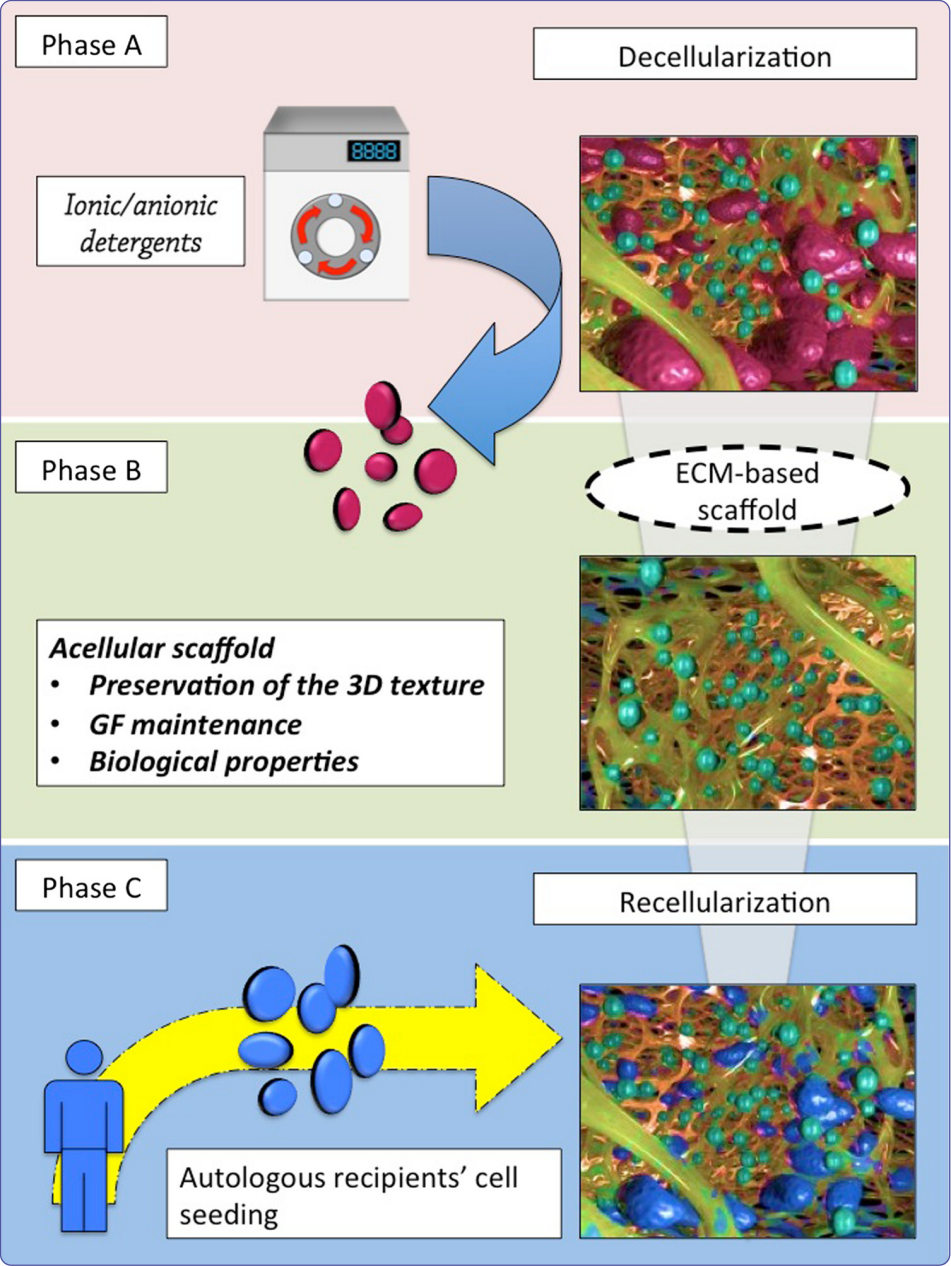
Key concepts of the tissue engineering and regenerative medicine paradigm. During the first step of the process (phase A), all the native cells are detached from the extracellular matrix (ECM) framework by using ionic and anionic detergents with different timings and concentrations. This procedure, called decellularization, produces an acellular ECM-based three-dimensional scaffold while keeping the native organ-specific structure almost intact. Phase B represents the second step, in which the scaffold is completely analyzed in order to check the effective preservation of the original texture, to quantify the growth factors present, and to study the scaffold’s biological properties. The last step is the seeding of the scaffold with organ-specific cells (phase C). In the best-case scenario, these cells come directly from the patient who will receive the bioengineered organ (autologous cells), avoiding immunological problems. This step, called recellularization, is a major obstacle to overcome due to the large number of cells needed to occupy the entire volume of the acellular scaffold. In addition to the number of cells, there is a need to maintain specific cell type proportions in order to establish a physiologically functional organ. Second, the exact cellular ‘cocktail’ for each organ needs to be established to get the perfect seeding in which all the cells are able to grow up autonomously once seeded. 3D, three-dimensional; GF, growth factor

Organ transplantation currently represents the gold-standard treatment for all diseases leading to irreversible organ failure [1]. Despite efforts to increase the supply pool of suitable organs for transplantation, a significant gap still exists between the numbers of organ donors and recipients, highlighting the major problem of organ shortage [2]. Tissue engineering and regenerative medicine (TE/RM) share the same ultimate target: the creation of functional tissues or whole organs and their use as ‘replacement parts’ for the human body [3]. Successful achievement of this goal will play a groundbreaking role in clinical transplantation [4]. A common approach of TE/RM is to create a structural and molecular environment that accurately mimics the properties (mechanical, geometrical, and biological) of the native organ in order to support the recipient’s cells and create an autologous tissue/organ. Although there have been several attempts to produce synthetic scaffolds, they have produced only constructs that partially mimic the natural vascular network. Recently, a new technology was introduced to overcome this problem by using whole-organ decellularization to create a three-dimensional (3D) extracellular matrix (ECM) that preserves the native tissue architecture, including the vasculature. Tissue decellularization is achieved by flushing the organ with detergent solutions through its native vascular system, which removes all native cell components while preserving the ECM molecules [5]. Researchers have used different detergents and techniques for tissue decellularization. Effective decellularization of whole organs depends on many factors, such as tissue density, thickness, and cellularity. All of the agents and protocols used for decellularization alter ECM composition and cause some disruption in the organ’s microarchitecture. Different agents that are often used for tissue decellularization include acids or bases, ionic (that is, sodium dodecyl sulphate, or SDS) and non-ionic (that is, Triton X-100) detergents, and enzymes (that is, trypsin) [5]. All of these agents have their advantages and disadvantages for specific tissue and organ decellularization because their mechanism of action is different. For example, Triton X is more effective on thinner tissue whereas SDS is more effective on thicker tissues. However, SDS is known to be very effective in cell removal but has a lesser degree of retention of various ECM molecules in the decellularized scaffold compared with a detergent such as Triton X-100. Chemical acid agents (that is, acetic acid or per-acetic acid) can solubilize the cytoplasmatic components removing the nucleic acids but, at the same time, they subtract the collagen from the matrix [6]. Biological agents are potential tools for decellularization. They can be divided into two main categories: enzymatic agents (that is, trypsin) and non-enzymatic agents (that is, ethylenediaminetetraacetic acid, or EDTA). Enzymatic agents can interrupt the protein-protein interaction with cellular detachment from ECM basal membrane but also damage the collagen structure of ECM. Non-enzymatic agents are able to unconnect the cells by separating their metal ions but are unsuccessful in cellular removal [7, 8]; for this reason, many decellularization protocols combine non-enzymatic biological agents with detergents able to gently remove deconnected cells from the matrix. Finally, physical strategies for decellularization involve freeze-thawing cycles and hydrostatic-based procedures. These methods can produce cellular lysis (and their subsequent removal from the ECM structure) but do great damage to the ECM architecture [7]. There are numerous methods of delivering the detergent to the tissues, such as perfusion or agitation. Specific to this review of whole organs, such as heart or liver, perfusion of detergent throughout the vasculature has proven to be the most effective in cell removal as well as maintenance of the organ’s microarchitecture [5, 9]. Although perfusion of detergent throughout the vasculature facilitates and increases cell removal from the organ, the pressure associated with perfusion could disrupt and create punctures within the vascular network; therefore, the flow rate in which the detergent is delivered to the organ also plays a critical role. ECM is the naturally occurring scaffold material secreted by the resident cells of each tissue and organ. The structural and functional molecules of the ECM are in a state of dynamic equilibrium with the surrounding tissue and provide the means by which cells communicate with each other and the tissue environment. The ECM contains growth factors and other bioinductive factors, which facilitate cell attachment, tissue integration, remodeling, and development [10, 11]. The ECM also provides organ-specific physical, biochemical, and biomechanical properties. The physical properties are important to stimulate anchorage-related biological function (cell division, tissue polarity, and cell migration) [12] and cellular mechanotransduction to convert the mechanical stimulus into chemical activity [13], whereas the biochemical properties provide local and soluble growth factor signals [10, 14]. Whole-organ decellularization provides an additional advantage for using this approach for preparation of scaffolds for tissue engineering. The preservation of the native vascular network, used to deliver the decellularization detergent, can be used to deliver cells to all areas of the scaffold and thereafter for efficient organ perfusion upon transplantation *in vivo*. In sum, the unique properties of the whole-organ ECM scaffold make it ideal for whole-organ bioengineering [15] (Fig. 1). Below, we provide a brief summary of TE/RM approaches for bioengineering of different organs using whole-organ ECM scaffolds.

## Liver bioengineering

The liver is the largest gland in the body and carries out numerous important functions. Some of these functions are metabolism; maintaining homeostasis; synthesis of amino acids, proteins, and enzymes; production of cholesterol and bile; and detoxification and elimination of drugs and harmful compounds. The liver also serves as an energy storage unit by storing fat and glycogen. The majority of these functions are carried out by hepatocytes, the major cell type in the liver, constituting about 70% to 80% of the total cell population in the liver. The liver is also made up of Kupffer cells, cholangiocytes, stellate cells, and sinusoidal endothelial cells, which work in harmony with the hepatocytes to carry out proper function of the liver. The liver has a natural ability to regenerate; it has been shown in mice that 70% to 80% of a healthy liver can be removed and the liver will still be able to carry out its function normally and grow. However, such is not the case for diseased livers [16]. Six hundred and fifty million people have liver disease worldwide and 21 million of these people have chronic liver disease [17]. In the US, 30 million people have liver disease [18]. Since there is a high prevalence of liver disease around the world and since transplantation is the only long-term treatment available, there is a great demand for livers. In the US, about 16,000 patients need a liver; however, only about 6,000 livers are transplanted every year, and 2,500 patients die waiting for liver donation since no other life-saving option is available [19]. Therefore, if TE/RM is successful, it can help solve the problem of liver shortage by increasing the number of organs that can be used for transplantation. Tissue decellularization using detergents such as Triton X or SDS has proven to be a successful method to prepare matrices and scaffolds for TE/RM [9, 20, 21].

There is an increased use of these decellularized, natural bioscaffolds because they not only maintain their microarchitecture but also retain many bioactive signals (cell-adhesion peptides, ECM proteins, and so on) that are difficult to replicate artificially and help with cell attachment and viability [22]. It is also advantageous to use whole-organ scaffolds because ECM components are consistent from species to species. Therefore, when it comes to humans, there is the potential to use decellularized porcine organs, since they are closest in size to human organs and are readily available [23, 24]. Human cells can be delivered to these decellularized porcine organs to generate bioengineered human organs [9]. In terms of liver bioengineering, it has been shown that natural matrices help with the growth and viability of primary hepatocytes [24]. Livers are decellularized by using the perfusion method because it has been the most effective in removal of cellular components of the organ and does little damage to the vascular network, two criteria which are extremely important in recellularization of the whole organ [25]. At present, several species of livers have been decellularized with different types of protocols to obtain natural bioscaffold [23, 26, 27]. In 2013, Kajbafzadeh and colleagues [28] reported the evaluation of two main decellularization techniques (diffusion and perfusion) and five different decellularization protocols for ovine livers. They determined that a perfusion method is a better decellularization technique, and that perfusion with ammonium hydroxide solution followed by cycles of Triton X-100 is the most accurate and appropriate decellularization protocol to obtain whole liver with an undamaged intravascular tree. The same method has been used in other studies as the current best liver-specific decellularization protocol [25]. In 2011, Baptista and colleagues [9] bioengineered a functional humanized rat liver by using a bioreactor system to deliver human progenitor cells to the liver scaffolds. The bioreactor provides a continuous flow of media with growth factors and gases which allows proper cell maintenance in the 3D liver scaffold. Different pressures can be used to deliver different cell populations to their appropriate niche in the liver. These bioengineered livers displayed hepatic characteristics such as biliary duct structures which were positive for cytokeratin 19 along with clusters of hepatocytes which were positive for cytochrome P450 3A and albumin in the parenchymal space of the liver. The bioengineered liver also displayed hepatic functions such as urea and albumin secretion along with the ability to metabolize drugs. The endothelial cells coated the liver vascular structures and expressed endothelial cell nitric oxide synthase. Furthermore, upon blood perfusion, there was significantly less platelet adhesion and aggregation in the bioengineered liver compared with that of the empty liver scaffold, which is an extremely important factor for blood vessel patency after transplantation. Scientists are attempting to use porcine liver as a scaffold for liver bioengineering since, as mentioned earlier, the porcine liver size is the closest in size to human livers [24]. There has been success in proper decellularization of porcine livers with maintenance of the vascular network and important ECM proteins; however, complete recellularization using all of the other liver cell types, including Kupffer, sinusoidal endothelial, and stellate cells, and bioengineering a fully functional liver which remains patent upon transplantation at a human liver scale have not yet been accomplished [23, 24]. One of the biggest challenges in whole-organ bioengineering is an appropriate cell source to repopulate a scaffold and this is no different for whole-liver bioengineering. In 2010, Espejel and colleagues [29] used induced pluripotent stem cell (iPSC) technology to create hepatocytes that have functional and proliferative capabilities for liver regeneration in mice. Using iPSC technology for liver cells provides a potential source of cells which could be used for eventual whole-liver bioengineering for humans since liver cells are extremely specialized cells. To date, no one has been able to isolate hepatocytes or liver endothelial cells and have them grow in culture in the long term [30, 31]. Upon isolation, these cells lose the capability to proliferate once outside of their natural environment. Espejel and colleagues showed liver regeneration after partial (two-thirds) hepatectomy in three wild-type and three FAH-deficient mice repopulated to approximately 100% with iPSC-derived hepatocytes. The iPSC source is a very promising cell source for liver regeneration as shown by Espejel and colleagues. In 2013, Takebe and colleagues [32] were the first to use iPSC technology to generate a 3D vascularized human liver *in vitro*. However, bioengineering a fully functional liver the size of a human liver has yet to be performed by using iPSC technology. Scientists have also looked into the use of progenitor cells to repopulate liver scaffolds; however, to get the appropriate cell numbers to bioengineer a liver to the size of a human liver remains an issue [9]. Both the iPSC technology and progenitor cells have their advantages and disadvantages. iPSCs have the advantage of being extremely proliferative and having an unlimited number of cell divisions; however, this can also be a disadvantage because unlimited cell divisions could give rise to tumors [33]. Progenitor cells have the advantage of being stem cell-like but also in a further stage of cell differentiation and have a limited number of cell divisions and therefore lack the ability to form tumors. Since progenitor cells have a limited number of cell divisions, it is extremely difficult to isolate a large number of these cells to repopulate a liver scaffold for liver transplantation. Therefore, since primary liver cells are extremely difficult to grow *in vitro*, the focus in the field of liver bioengineering needs to go toward generating billions of the specialized liver cells (hepatocytes, stellate cells, sinusoidal endothelial cells, and so on) to bioengineer a transplantable human liver for patients with liver disease.

## Kidney bioengineering

In the US, approximately 1 million patients live with end-stage renal disease (ESRD), and there are over 100,000 new diagnoses every year. Although hemodialysis has increased the survival of patients with ESRD, kidney transplantation remains the only potential curative treatment. Despite the advances in renal transplant immunology, 20% of recipients will experience an episode of acute rejection within 5 years of transplantation, and approximately 40% of recipients will die or lose graft function within 10 years. The limitations of current therapies for renal failure have led researchers to explore the development of alternative modalities that could improve, restore, or replace either partial or total renal function [34–37]. Owing to the unique anatomy and physiology of the kidney, whole-kidney ECM scaffolds are a potentially groundbreaking approach for kidney bioengineering. In this endeavor, several decellularization protocols using different types of detergents and enzymes have been described. The perfusion through the kidney vasculature is an efficient method for delivering detergents to cells and for removal of cellular material from the tissue. However, their effects on kidney microstructure have not been studied extensively [5, 38, 39]. Recently, Caralt and colleagues [40] published research that represents the state of the art about the optimization of the decellularization procedure for rat kidney. Three strategies of cellular removal have been analyzed (perfusion with Triton X-100 alone, sequential perfusion of 1% Triton X-100 and 0.1% SDS, and sequential perfusion with 0.02% Trypsin and 0.05% EDTA/Triton X-100 solution) evaluating the effective cellular removal from kidneys and the preservation of the native architecture and of the original biological properties of the organ. Their conclusion was that Triton/SDS was the most efficient strategy to decellularize rat kidneys while maintaining a balance between the cellular removal and the conservation of the original architecture, of the major ECM proteins, and of the growth factors [40]. The kidney has approximately 30 different specialized cell types, including approximately 2 million glomeruli, and a complex network of arteries, veins, and capillaries. To bioengineer an efficient and functional kidney, all cell types must be present and viable, and this represents a major challenge [41, 42]. Several efforts have been performed to identify a reliable cell source for kidney recellularization, including adult kidney cells, mesenchymal and bone marrow stem cells, and iPSCs [43–47]. Harari-Steinberg and colleagues [48] identified nephron progenitor cells in human kidneys, which were capable of generation of kidney structures and functional repair of chronic renal disease. These cells expressed NCAM1^+^ and had a high clonogenic potential. When these cells were grafted in aggregates into a chorioallantoic membrane of the chick embryo, they generated renal structures [48]. Human amniotic stem cells (HASCs) express surface markers and transcription factors distinctive of embryonic stem cells (ESCs). These include octamer-binding transcription factor 4 (OCT-4) and stage-specific embryonic antigen-4 (SSEA-4). HASCs have high replicative self-renewal potential and multilineage differentiation capacity. Perin and colleagues [49] showed that HASCs integrated into metanephric structures after being injected into embryonic kidneys, which improved repair/recovery of kidneys with acute tubular necrosis [50]. iPSCs were first described by Takahashi and Yamanaka [51] in 2006, when they reprogrammed human fibroblasts to become pluripotent stem cells by the addition of four different genes: *Oct3/4*, *Sox2*, *c-Myc*, and *Klf4*. Despite being a good source of cells, not all adult stem cells can be reprogrammed by using the same method, which means that each cell type may have critical factors. Unlike ESCs, iPSCs have no ethical issues and no immune rejection. The surrogate application of iPSCs as representative of kidney disease is increasingly becoming reality given recent advances involving the production of iPSCs from both mesangial and epithelial cells derived from urine [52]. Song and colleagues [53] used human umbilical vein endothelial cells, delivered through the artery, for re-endothelialization and neonatal rat kidney cells, delivered through the ureter, for whole rat kidney bioengineering. Scanning electron microscopy of reseeded kidneys showed perfused glomerular capillaries with engrafted podocytes and formation of foot processes [53]. Future directions for kidney bioengineering are renal progenitor cell isolation, differentiation, expansion, and optimization of cell seeding protocols and culture.

## Pancreas bioengineering

Diabetes mellitus type 1 represents a global disease with more than 280 million patients worldwide [54]. Its therapy is focused principally on life-long insulin treatment, which does not provide a complete cure [55]. Beta-cell replacement is the only definitive treatment for type 1 diabetes since it is the only way to achieve glucose-responsive insulin secretion to ensure euglycemia. Unfortunately, islets are very sensitive to the hypoxic environment that they encounter during the process of islet isolation and transplantation as well as the immunological rejection of donor islets even in the presence of immunosuppressive therapy [56]. Regenerative medicine, and particularly whole-organ engineering, may offer some solutions to these outstanding challenges, as we describe below. Pancreas bioengineering is based on the use of pancreatic ECM, obtained by detergent-based decellularization techniques, as a two-dimensional and 3D scaffolding system for islet seeding and delivery. The pancreas-specific ECM preserves the native tissue morphology and biological properties and can support islet cell viability and survival [57–59] because of its capacity to maintain active pancreas-specific growth factors [60–62]. This technique also preserves the native vascular network, important for subsequent *in vivo* pancreas transplantation. De Carlo and colleagues [63] reported that pancreatic ECM supported islet survival and functionality in a synthetic device. In a recent study, Goh and colleagues [62] showed the ability to create acellular rat whole-pancreas scaffolds and reseed them with a beta-cell line. Recently, major efforts have focused on developing animal models, particularly pigs, in order to demonstrate long-term viability and function of clinical-size bioengineered pancreata. Mirmalek-Sani and colleagues [64] created an intact pancreas ECM scaffold by using a detergent-based infusion technique. These scaffolds were subsequently seeded with pancreatic islets and showed insulin secretion by seeded islets [64]. Moreover, the decellularization protocol proposed in this article (whole-organ perfusion with Triton X-100 and DNase-based solutions) currently represents the most suitable decellularization technique to achieve a clinical-size pancreatic acellular scaffold. In fact, this strategy can remove cells from pancreatic tissue without destroying either the essential ECM proteins (collagen, elastin, fibronectin, and laminin) or its precise 3D organization. Even though a human-scale completely functional bioengineered pancreas has not yet been achieved, these recent results represent a viable approach that can be combined with stem cells and iPSCs to obtain a transplantable bio-pancreas.

## Airway bioengineering

In 2013, 1,923 lung transplants were performed for several disorders, including congenital diseases, cystic fibrosis, emphysema/chronic obstructive pulmonary disease, alpha-1-antitrypsin deficiency, primary pulmonary hypertension, and other disorders (like sarcoidosis, bronchiectasis, and pulmonary vascular disease) [65]. Airway tissue engineering has the potential to augment patient survival and to reduce the waiting list for lung transplantation. TE/RM has only recently targeted lungs, whereas, previously, upper airway tracts have been the focus and were introduced into clinical practice [66, 67]. In fact, within organ bioengineering, the airway has been one of first organs to achieve an extraordinary result in the clinical setting; in 2008, Macchiarini and colleagues [68] performed the first bioengineered trachea transplantation in human patients. Airway bioengineering represents a very appealing alternative to ‘orthodox’ reconstructive techniques using autologous or allogenic tissues, but to understand the real challenge in this specific area, it is mandatory to highlight that all airway structures have a two-sided organization: one is directly in contact with the external environment, whereas the second is linked to the body. This particular configuration makes it essential to build a perfectly functioning bioengineered airway organ. Tracheas were the first step in this specific field and recently were followed by important published articles on bioengineered larynx and lungs [69, 70]. Compared with lungs, trachea and larynx offer different solutions regarding scaffolds to recellularization. Owing to their relatively simple hollow shape, several synthetic or semi-synthetic alternatives have been examined. Synthetic scaffolds have been tested as first attempts to integrally replace the trachea because of their advantageous characteristics: they do not need a donor, they can be easily modified to recipient conformation, and finally they can be sterilized and thus avoid the risk of post-transplantation infection. On the other hand, synthetic scaffold use is limited by several factors, including a low level of integrity, different mechanical properties compared with the native structure, a propensity to infective contamination, and non-vascularization [71]. The absence of vascularization represents the major issue to resolve as synthetic scaffolds cause apoptosis of all types of cells eventually seeded on them [72]. The most relevant materials tested for this purpose have been polyester urethane, polypropylene mesh, alginate gel, polyethylene glycol-based hydrogel, and poly-e-caprolactone [73]. Semi-synthetic scaffolds represent the evolution of synthetic scaffolds. They have been built by using a combination of natural and synthetic materials in the same assembly. Macchiarini’s group used this solution, based on cell seeding on a collagen-coated polypropylene scaffold, to perform a tracheobronchial transplantation in 2011 [74]. Nonetheless, the interplay between cells and scaffolds (even if semi-synthetic) is crucial for the correct cell-to-cell interaction as well as for cell migration and differentiation and needs to be considered for any clinical translation. Natural scaffolds obtained by a decellularization technology are the most promising outcomes in organ bioengineering. Several methods have been used to remove cells and achieve acellular ECM-based scaffolds with all the major properties that cells need for tissue or organ regeneration. At present, only one method clinically accepted for tracheal bioengineering [75] can manufacture an acellular non-immunogenic 3D ECM scaffold preserving most of the biological and mechanical qualities of the native trachea. These characteristics make this scaffold perfectly suitable for cell seeding. The use of human tissues and organs for decellularization might raise the immunogenicity issue associated with human donor-derived materials. Using animal organs can overcome this limitation, but xenogenic platforms have not yet been translated to the clinical setting. For either scenario (animal or human), the length of decellularization methods introduces great risk of contamination [72]. Although decellularization protocols can cause loss of glycosaminoglycans and other ECM elements, this technology remains the best choice to obtain a 3D scaffold to repopulate preserving, as the clinical practice showed, the adequate properties for a correct cellular long-term maturation. Two principal cell types are required to recellularize upper airway scaffolds: chondrocytes and epithelial cells. For both kinds of cells, several strategies have been tested, although the best solution for harvesting and reseeding them has yet to be standardized. Theoretically, epithelial cells are ready to harvest in the form of nasal epithelia but *in vivo* they do not show abilities to be stratified and then recreate the trachea-specific pseudo-stratified columnar epithelium [76]. Different sources of cells have been investigated to discover the optimal solution for recellularization, including iPSCs (that have been differentiated into functional airway and lung epithelium [77–79]), bone marrow-derived hematopoietic progenitor cells [80], human ESCs [81], and amniotic fluid-derived stem cells [82]. Even if laryngeal bioengineering is a more challenging field for regenerative medicine, owing principally to the incredible complexity of laryngeal anatomy, some interesting results have been achieved. In 2011, Baiguera and colleagues [69] developed a human laryngeal scaffold by using a detergent-enzymatic-based decellularization protocol. That scaffold was characterized by the preservation of all the structures composing the larynx and of its biomechanical properties. This result suggests that the creation of a transplantable bioengineered larynx is feasible although neovascularization and nervous innervation will be hard problems to solve. Production of bioengineered lungs for total organ replacement could be defined as the final goal for respiratory regenerative medicine. In 2010, Petersen and colleagues [70] showed how rat lungs can be decellularized leaving ECM-based scaffolds. These structures have been seeded in a specific bioreactor and then transplanted in recipient rats for a short period (45 to 120 minutes). Results indicated that the bioengineered lung participated in gas exchange [70]. From this model, other decellularization methods have been translated to large animal models, including rhesus monkey [83, 84], pig, and human [66, 85]. Compared with the trachea or larynx, the lung has a more complex 3D structure and recellularization approaches are challenging, including the selection of the best cell type for repopulation. A complete recellularization protocol must involve epithelial and endothelial cells. Alveolar epithelial compartments could be principally divided into alveolar epithelial type I and type II (AETI and II) cells. In 2010, Price and colleagues [86] showed that decellularized lung ECMs can sustain the growth of fetal AETII cells, indicating that the ECM retains all the components that cells needed for the differentiation even after the cellular removal. Endothelial cell compartments can be repopulated by using different strategies including the use of ESCs [87, 88], mesenchymal stem cells [89, 90], and fetal or neonatal lung cells [91]. Decellularized lung scaffolds seem to be among the most promising tools to optimize in order to achieve transplantable cutting-edge bioengineered lungs. Moving toward this goal requires standardizing a decellularization protocol for the production of clinical-scale acellular scaffolds and optimizing recellularization methods to acquire a complete and homogeneous cellular distribution.

## Heart bioengineering

In the US, more than 4,000 people are waiting for a cardiac donation [36] and around 6 million persons are living with heart failure. A regenerative medicine approach of heart bioengineering could provide a theoretically unlimited source of replacement organs. Acellular heart scaffold could be identified as the paradigm for decellularization/recellularization technology as one of the first ECM-based platforms obtained by using whole-organ cellular removal via detergent perfusion. In 2008, Ott and colleagues [20] showed the huge potential of regenerative medicine and organ bioengineering by growing a beating heart in the laboratory. In their report, they demonstrated how a rat heart could be completely decellularized by perfusing with specific detergents (Triton X-100 and SDS) nearly preserving its native morphological, biological, and mechanical properties. They also reseeded the cardiac ECM with neonatal cardiac cells showing, after 8 days of culturing, persistency of contractility and beating under a specific electrical impulse. This pioneering study drove several groups to successfully decellularize/recellularize the heart in rodent models [92–94] with similar results. Although several protocols were tested, there is no evidence for any definitive one at present. This model has been scaled up to the large animal that provides a relevant and translational clinical size [95, 96]. Many groups obtained 3D heart scaffolds by using similar detergents and concentrations just augmenting the time of their exposure to cells. In 2010, Wainwright and colleagues [95] developed a further method to decellularize an entire heart, obtaining a 3D acellular cardiac organoid with a microenvironment that supported site-appropriate cell differentiations. Two years laterbased on this protocol, Remlinger and colleagues [96] used a retrograde-perfusion protocol for whole-heart decellularization. In that article, they created a slightly more aggressive protocol that led to a reduction of residual DNA in the scaffold. This result has to be carefully analyzed because no beneficial effects on recellularization had ever been demonstrated for such a low quantity of DNA, as reviewed by Momtahan and colleagues [97]. Reasonably, a balance between aggressive decellularization protocols and maintenance of the microenvironment could be positive for recellularization and still needs to be more deeply investigated. Recently, Methe and colleagues [98] proposed an alternative procedure to decellularize porcine hearts by using hypotonic solution and ionic and non-ionic detergents (4% sodium deoxycholate and 1% Triton X-100, respectively) via perfusion and agitation methods. The resultant ECM preserved its native morphological and functional integrity. Interestingly, this protocol does not affect the cardiomyocyte cytoskeleton that remains almost intact. In cardiac regenerative medicine, different cellular families had been considered. Cellular type is just a single ingredient for the final result that (to be successful and functional) should include the provision of specific growth factors and nutrients inside a dedicated bioreactor. The most important cell types that have been considered for cardiac bioengineering are ESCs [99–101] and iPSCs [95, 102, 103]. Even if iPSCs have the potential to be considered the best solution to repopulate an acellular cardiac scaffold, some issues still need to be resolved: improving their effectiveness of dedifferentiation, removing the risk of teratoma development, improving culture techniques, and enhancing new strategies for their distribution into acellular scaffolds [97]. Finally, growth factor addition has to be taken into consideration for efficient recellularization. Many growth factors can be used in cardiac bioengineering, and the most significant are bone morphogenetic protein (BMP) [104], basic fibroblast growth factor (bFGF) [105], BMP-4 [93], and vascular endothelial growth factor (VEGF) [106]. In 2007, Van Wijk and colleagues [104] summarized how BMPs are crucial for cardiac differentiation (and for dedifferentiation starting from iPSCs) not only in specific heart-forming regions but also at cardiac distal margins. Perets and colleagues [105] demonstrated how bFGF could stimulate angiogenesis inducing the proliferation of endothelial cells, smooth muscle cells, and fibroblast on alginate scaffolds. VEGF has been identified as one of the major stimuli for angiogenesis (*in vitro* and *in vivo*) that actually remains a big challenge to address limiting organ bioengineering. Zisch and colleagues [106] created a new form of synthetic matrices that incorporate covalent variants of VEGF (VEGF121 and VEGF165). After implantation, VEGF-containing matrix was adapted into native vascularized tissue.

## Summary

In recent years, several bioengineered tissues have been created and transplanted in humans. These were relatively simple structures such as blood vessels, upper airway tubes, or urogenital tissues. The larger challenge, however, remains the bioengineering of complex parenchymal organs (for example, the kidney or liver) for human transplantation. In 2011, almost 3,800 human organs, originally destined for transplant, were discarded for various reasons [107] (Table 1). Even though this number of organs represents only about 13.5% of all donated organs, it represents a tragic waste of material that could have been used in organ bioengineering investigations. As recent reports have shown, decellularization/recellularization techniques can be applied to human organs such as kidneys [108], lungs [66, 109], and small intestine [110] and consequently can serve as a platform for TE/RM. However, a major challenge still exists in the complete repopulation of these whole-organ scaffolds, which is necessary to produce a clinically functional organ. Identification of a cell source that has the potential to proliferate after scaffold seeding may offer a solution. Furthermore, even if the whole-organ ECM scaffold was made from animal tissue, their species-specific biological and biomechanical properties are suitable for human cell seeding. Lastly, the use of discarded human organs, with a complete patient history, can facilitate regulatory approval of these scaffolds for clinical use.

**Table 1:** Statistical data on organ transplantation in the US for 2012

	Recovered	Transplanted	Discarded	Loss rate, percentage
Pancreas	1,562	1,143	419	26.8
Kidney	14,784	12,140	2,644	17.9
Liver	6,685	6,030	655	9.8
Intestines	136	129	7	5.2
Lungs	3,302	3,163	139	4.2
Heart	2,382	2,365	17	0.7
All organs	28,851	24,970	3,881	13.5

Data show how yearly almost 4,000 organs, originally destined for transplant, are discarded for different reasons. These organs could represent a unique source for regenerative medicine and organ bioengineering research. Source: *New York Times* [107].

## Conclusions

Whole-organ bioengineering using ECM scaffolds offers several advantages over ‘classic’ synthetic scaffold:

- They provide a natural environment for seeded cells, similar to the native organ, and include organ-specific biochemical stimuli such as growth factors, cytokines, or chemokines.
- They maintain the original 3D architecture after decellularization. This could support the progressive process of tissue engineering that includes cell seeding on the scaffold ⇒ attachment ⇒ growth ⇒ expansion ⇒ maturation.
- They can be transplanted *in vivo* via a dual vascular pedicle (arterial and venous), guaranteeing physiological oxygen and nutrient supply.

Although major advances were made recently in the field of TE/RM toward the bioengineering of transplantable organs, many challenges remain (Table 2). These include the determination of specific criteria for successful decellularization, identification of a reliable cell source for the recellularization, and the development of models for bioengineered organ transplantation with long-term follow-up studies that can translate into clinical practice.

**Table 2:** Limiting factors for improving the decellularization/recellularization technology

Topic of interest	Primary issue	Research goals
Protocols of decellularization	Standardization of decellularization methods for each organ	Achievement of a reproducible method to obtain scaffold from different organs and different species
Cell source	Identification of suitable cell source	Recellularization of whole-organ scaffolds
	Autologous cells	
	Heterologous cells	
Large animal model	Creation of models for transplantation with a long-term functional follow-up	Obtain organs for human clinical transplantation

1. Standardization of cellular removal protocols for each organ.

2. The identification of the most suitable cell source for the most effective seeding.

3. The creation of a large animal model to standardize the implantation techniques limiting eventual side effects.

## Note

This article is part of a thematic series on *Functional imaging in regenerative medicine*. Other articles in this series can be found at http://stemcellres.com/series/FIRM.

## Abbreviations

3D: three-dimensional
AET: alveolar epithelial type
bFGF: basic fibroblast growth factor
BMP: bone morphogenetic protein
ECM: extracellular matrix
EDTA: ethylenediaminetetraacetic acid
ESC: embryonic stem cell
ESRD: end-stage renal disease
HASC: human amniotic stem cell
iPSC: induced pluripotent stem cell
SDS: sodium dodecyl sulphate
TE/RM: tissue engineering and regenerative medicine
VEGF: vascular endothelial growth factor

## References

[1] World Health Organization (2013). World Health Statistic.

[2] United Network for Organ Sharing. http://www.unos.org. Accessed April 2014.

[3] Atala A (2007). Engineering tissues, organs and cells. J Tissue Eng Regen Med.

[4] Orlando G, Soker S, Stratta RJ (2013). Organ bioengineering and regeneration as the new Holy Grail for organ for organ transplantation. Ann Surg.

[5] Crapo PM, Gilbert TW, Badilak SF (2011). An overview of tissue and whole organ decellularization processes. Biomaterials.

[6] Reing JE, Zhang L, Myers-Irvin J, Cordero KE, Freytes DO, Heber-Katz E (2009). Degradation products of extracellular matrix affect cell migration and proliferation. Tissue Eng Part A.

[7] Brown BN, Freund JM, Li H, Rubin JP, Reing JE, Jeffries EM (2011). Comparison of three methods for the derivation of a biologic scaffold composed of adipose tissue extracellular matrix. Tissue Eng Part C.

[8] Khan AA, Vishwakarma SK, Bardia A, Venkateshwarulu K (2014). Repopulation of decellularized whole organ scaffold using stem cells: an emerging technology for the development of neo-organ. J Artif Organs.

[9] Baptista PM, Siddiqui MM, Lozier G, Rodriguez SR, Atala A, Soker S (2011). The use of whole organ decellularization for the generation of vascularized liver organoid. Hepatology.

[10] Hynes RO (2009). The extracellular matrix: not just pretty fibrils. Science.

[11] Watt FM, Huck WT (2013). Role of the extracellular matrix in regulating stem cell fate. Nat Rev Mol Cell Biol.

[12] Lu P, Weaver VM, Werb Z (2012). The extracellular matrix: a dynamic niche in cancer progression. J Cell Biol.

[13] DuFort CC, Paszek MJ, Weaver VM (2011). Balancing forces: architectural control of mechanotransduction. Nat Rev Mol Cell Biol.

[14] Gattazzo F, Urciolo A, Bonaldo P (2014). Extracellular matrix: a dynamic microenvironment for stem cell niche. Biochim Biophys Acta.

[15] Soto-Gutierrez A, Wertheim JA, Ott HC, Gilbert TW (2012). Perspective on whole-organ assembly: moving toward transplantation on demand. J Clin Invest.

[16] Higgens G, Anderson R (1931). Experimental pathology of the liver: restoration of liver of the white rat following partial surgical removal. Arch Pathol Lab Med.

[17] World Health Organization (2014). World Health Statistics.

[18] Chronic Liver Disease and Cirrhosis. Vol. 2014 (Centers for Disease Control and Prevention). http://www.cdc.gov/nchs/fastats/liver-disease.htm. Accessed 25 Jun 2014.

[19] Waiting List Candidates. Vol. 2014 (Organ Procurement and Transplantation Network). http://optn.transplant.hrsa.gov. Accessed 25 Jun 2014.

[20] Ott HC, Matthiesen TS, Goh SK, Black LD, Kren SM, Netoff TI (2008). Perfusion-decellularized matrix: using nature’s platform to engineer a bioartificial heart. Nat Med.

[21] Badylak SF (2007). The extracellular matrix as a biologic scaffold material. Biomaterials.

[22] Kim BS, Baez CE, Atala A (2000). Biomaterials for tissue engineering. World J Urol.

[23] Lin P, Chan WC, Badilak SF, Bhatia SN (2004). Assessing porcine liver-derived biomatrix for hepatic tissue engineering. Tissue Eng.

[24] Yagi H, Fukumitsu K, Kukuda K, Kitago M, Shinoda M, Obara O (2013). Human-scale whole-organ bioengineering for liver transplantation: a regenerative medicine approach. Cell Transplant.

[25] Ko IK, Peng L, Peloso A, Smith CJ, Dhal A, Deegan DB (2015). Bioengineered transplantable porcine livers with re-endothelialized vasculature. Biomaterials.

[26] Shupe T, Williams M, Brown A, Willemberg B, Petersen BE (2010). Method for the decellularization of intact rat liver. Organogenesis.

[27] Gilbert TW, Agragal V, Gilbert MR, Povirk KM, Badylak SF, Rosen CA (2009). Liver-derived extracellular matrix as a biologic scaffold for acute vocal fold repair in a canine model. Laryngoscope.

[28] Kajbafzadeh AM, Javan-Farazmand N, Monajemzadeh M, Baghayee A (2013). Determining the optimal decellularization and sterilization protocol for preparing a tissue scaffold of a human-sized liver tissue. Tissue Eng Part C.

[29] Espejel S, Roll GR, McLaughlin KJ, Lee AY, Zhang JY, Laird DJ (2010). Induced pluripotent stem cell-derived hepatocytes have the functional and proliferative capabilities needed for liver regeneration in mice. J Clin Invest.

[30] Miranda JP, Leite SB, Muller-Vieira U, Rodrigues A, Carrondo MJ, Alves PM (2009). Toward an extended functional hepatocyte in vitro culture. Tissue Eng Part C Methods.

[31] March S, Hui EE, Underhill GH, Khetani S, Bhatia SN (2009). Microenvironment regulation of the sinuosoidal endothelial cell phenotype in vitro. Hepatology.

[32] Takebe T, Sekine K, Enomura M, Koike H, Kimura M, Ogaeri T (2013). Vascularized and functional human liver from an iPSC-derived organ bud transplant. Nature.

[33] Ebben JD, Zorniak M, Clark PA, Kuo JS (2011). Introduction to induced pluripotent stem cells: advancing the potential for personalized medicine. World Neurosurg.

[34] Wolfe RA, Ashby VB, Milford EL, Ojo AO, Ettenger RE, Agodoa LY (1999). Comparison of mortality in all patients on dialysis, patient on dialysis awaiting transplantation, and recipients of a first cadaveric transplant. N Engl J Med.

[35] Abecassis M, Barlett ST, Collins AJ, Davis CL, Delmonico FL, Friedewald JJ (2008). Kidney transplantation as primary therapy for end-stage renal disease: a National Kidney Foundation/Kidney Disease Outcomes Quality Initiative (NKF/KDOQITM) conference. Clin J Am Soc Nephrol.

[36] Organ Procurement and Transplantation Network: United Network for Organ Sharing 2012 Annual Report: Health Resources and Services Administration. Richmond, US Department of Health and Human Services, 2012. http://www.unos.org/docs/AnnualReport2012.pdf. Accessed April 2014.

[37] Katari R, Peloso A, Zambon JP, Soker S, Stratta RJ, Atala A (2014). Renal bioengineering with scaffolds generated from human kidneys. Exp Nephrol.

[38] Faulk DM, Carruthers CA, Warner HJ, Kramer CR, Reing JE, Zhang L (2014). The effect of detergents on the basement membrane complex of a biologic scaffold material. Acta Biomater.

[39] Sullivan DC, Sayed-Hadi MS, Deegan DB, Baptista PM, Aboushwareb T, Atala A (2012). Decellularization methods of porcine kidneys for whole organ engineering using a high-throughput system. Biomaterials.

[40] Caralt M, Uzarski JS, Iacob S, Obergfell KP, Berg N, Bijonowski BM (2014). Optimization and critical evaluation of decellularization strategies to develop renal extracellular matrix scaffolds as biological templates for organ engineering and transplantation. Am J Transplant.

[41] Vorotnika E, McIntosh D, Dewilde A, Zhang J, Reing JE, Zhang L (2010). Extracellular matrix-derived products modulate endothelial and progenitor cell migration and proliferation in vitro and stimulate regenerative healing in vivo. Matrix Biol.

[42] Bornstein P, Sage EH (2002). Matricellular proteins: extracellular modulators of cell function. Curr Opin Cell Biol.

[43] Al-Awqati Q, Oliver JA (2006). The kidney papilla is a stem cell niche. Stem Cell Rev.

[44] Franquesa M, Flaquer M, Cruzado JM, Grinyò JM (2013). Kidney regeneration and repair after transplantation. Curr Opin Organ Transplant.

[45] Sagrinati C, Netti GS, Mazzinghi B, Lazzeri E, Liotta F, Frosali F (2006). Isolation and characterization of multipotent progenitor cells from the Bowman’s capsule of adult human kidneys. J Am Soc Nephrol.

[46] Angelotti ML, Ronconi E, Ballerini L, Peired A, Mazzinghi B, Sagrinati C (2012). Characterization of renal cell progenitors committed toward tubular and their regenerative potential in renal tubular injury. Stem Cells.

[47] Bonandrini B, Figliuzzi M, Papadimou E, Morigi M, Perico N, Casiraghi F (2014). Recellularization of well-preserved acellular kidney scaffold using embryonic stem cells. Tissue Eng Part A.

[48] Harari-Steinberg O, Metsuyanim S, Omer D, Gnatek Y, Gershon R, Pri-Chen S (2013). Identification of human nephron progenitors capable of generation of kidney structures and functional repair of chronic renal disease. EMBO Mol Med.

[49] Perin L, Giuliani S, Jin D, Sedrakyan S, Carraro G, Habibian R (2007). Renal differentiation of amniotic fluid stem cells. Cell Prolif.

[50] Zambon JP, Magalhaes RS, Ko I, Ross CL, Orlando G, Peloso A (2014). Kidney regeneration: where we are and future perspective. World J Nephrol.

[51] Takahashi K, Yamanaka S (2006). Induction of pluripotent stem cells from mouse embryonic and adult fibroblast culture by defined factors. Cell.

[52] Zhou T, Benda C, Dunzinger S, Huang Y, Ho JC, Yang J (2012). Generation of human induced pluripotent stem cells from urine sample. Nat Protoc.

[53] Song JJ, Guyette JP, Gilpin SE, Gonzalez G, Vacanti JP, Ott HC (2013). Regeneration and experimental orthotopic transplantation of a bioengineered kidney. Nat Med.

[54] Kumar SP, Adhikari P, Jeganathan PS, Sisodia V (2013). Exploring the research on diabetes mellitus: status of current evidence from a 40 year quantitative tend analysis of published articles. Int J Health Rehabil Sci.

[55] Bloomgarden ZT (2004). Diabetes complications. Diabetes Care.

[56] Orlando G, Gianello P, Salvatori M, Stratta RJ, Soker S, Ricordi C (2014). Cell replacement strategies aimed at reconstruction of the β-cell compartment in type 1 diabetes. Diabetes.

[57] Lucas-Clerc C, Massert C, Campion JP, Campion JP, Launois B, Nicol M (1993). Long-term culture of human pancreatic islets in an extracellular matrix: morphological and effects. Mol Cell Endocrinol.

[58] Salvay DM, Rives CB, Zhan X, Chen F, Kaufman DB, Lowe WL (2008). Extracellular matrix protein-coated the reversal of diabetes after extrahepatic islet transplantation. Transplant.

[59] Zhang Y, Jalili RB, Warnock GL, Ao Z, Marzban L, Ghahary A (2012). Three-dimensional scaffolds reduce islets amyloid formation and enhance survival and function of cultured human islets. Am J Pathol.

[60] Parnaud G, Hammar E, Ribaux P, Donath MY, Berney T, Halban PA (2009). Signaling implicated in the stimulation of beta-cell proliferation by extracellular matrix. Mol Endocrinol.

[61] Beattie GM, Rubin JS, Mally MI, Otonkoski T, Hayek A (1996). Regulation of proliferation and differentiation of human fetal pancreatic islet cells by extracellular matrix, hepatocyte growth factor, and cell-cell contact. Diabetes.

[62] Goh S-K, Bertera S, Olsen P, Candiello JE, Halfter W, Uechi G (2013). Perfusion-decellularized pancreas as a natural 3D scaffold for pancreatic tissue and whole organ engineering. Biomaterials.

[63] De Carlo E, Baiguera S, Conconi MT, Vigolo S, Grandi C, Lora S (2010). Pancreatic acellular matrix supports islet survival and function in a synthetic tubular device: in vitro and vivo studies. Int J Mol Med.

[64] Mirmalek-Sani SH, Orlando G, McQuilling JP, Pareta R, Mack DL, Salvatori M (2013). Porcine pancreas extracellular matrix as a platform for endocrine pancreas bioengineering. Biomaterials.

[65] Organ Procurement and Transplantation Network. http://optn.transplant.hrsa.gov. Accessed April 2014.

[66] Gilpin SE, Guyette JP, Gonzalez G, Ren X, Asara JM, Mathisen DJ (2013). Perfusion decellularization of human and porcine lungs: bringing the matrix to clinical scale. J Heart Lung Transplant.

[67] Gonfiotti A, Jaus MO, Barale D, Baiguera S, Comin C, Lavorini F (2014). The first tissue-engineered airway transplantation: 5-year follow-up results. Lancet.

[68] Macchiarini P, Jungebluth P, Go T, Asnaghi MA, Rees LE, Cogan TA (2008). Clinical transplantation of a tissue-engineered airway. Lancet.

[69] Baiguera S, Gonfiotti A, Jaus M, Comin CE, Paglierani M, Del Gaudio C (2011). Development of bioengineered human larynx. Biomaterials.

[70] Petersen TH, Calle EA, Zhao L, Lee EJ, Gui L, Raredon MB (2010). Tissue-engineered lungs for in vivo implantation. Science.

[71] Grillo HC (2002). Tracheal replacement: a critical review. Ann Thorac Surg.

[72] Jungebluth P, Moll G, Baiguera S, Macchiarini P (2012). Tissue-engineered airway: a regenerative solution. Clin Pharm Ther.

[73] Kucera KA, Doss AE, Dunn SS, Clemson LA, Zwischenberger JB (2007). Tracheal replacements: part 1. ASAIO J.

[74] Jungebluth P, Alici E, Baiguera S, Le Blanc K, Blomberg P, Bozóky B (2011). Tracheobronchial transplantation using a stem cell-seeded bioartificial nanocomposite: a proof-of-concept study. Lancet.

[75] Baiguera S, Damasceno KL, Macchiarini P, Lee C, Uygun K (2010). Detergent-enzymatic method for bioengineering human airways. Organ Perfusion and Culture Methodology. Methods in Bioengineering.

[76] Berg M, Ejnell H, Kovàcs A, Nayakawde N, Patil PB, Joshi M (2013). Replacement of a tracheal stenosis with a tissue-engineered human trachea using autologous stem cells: a case report. Tissue Eng Part A.

[77] Ghaedi M, Mendez JJ, Bove PF, Sivarapatna A, Raredon MS, Niklason LE (2014). Alveolar epithelial differentiation of human induced pluripotent stem cells in a rotating bioreactor. Biomaterials.

[78] Wong AP, Bear CE, Chin S, Pasceri P, Thompson TO, Huan LJ (2012). Directed differentiation of human pluripotent stem cells into mature airway epithelia expressing functional CFTR protein. Nat Biotechnol.

[79] Firth A, Dargotz CT, Qualls SJ, Menon T, Wright R, Singer O (2014). Generation of multiciliated cells in functional airway epithelia from human induced pluripotent stem cells. Proc Natl Acad Sci U S A.

[80] Seguin A, Baccari S, Holder-Hespinasse M, Bruneval P, Carpentier A, Taylor DA (2013). Tracheal regeneration: evidence of bone marrow mesenchymal stem cell involvement. J Thorac Cardiovasc Surg.

[81] McIntyre BA, Aler C, Mechael R, Salci KR, Lee JB, Fiebig-Comyn A (2014). Expansive generation of functional airway epithelium from human embryonic stem cells. Stem Cells Transl Med.

[82] Li Y, Xu W, Yan J, Xia Y, Gu C, Ma Y (2014). Differentiation of human amniotic fluid-derived mesenchymal stem cells into type II alveolar epithelial cells in vitro. Int J Mol Med.

[83] Bonvillain RW, Danchuk S, Sullivan DE, Betancourt AM, Semon JA, Eagle ME (2012). A nonhuman primate model of lung regeneration: detergent-mediated decellularization and initial in vitro recellularization with mesenchymal stem cells. Tissue Eng Part A.

[84] Nakayama KH, Lee CC, Batchelder CA, Tarantal AF (2013). Tissue specificity of decellularized rhesus monkey kidney and lung scaffolds. PLoS One.

[85] O’Neill JD, Anfang R, Anandappa A, Costa J, Javidfar J, Wobma HM (2013). Decellularization of human and porcine lung tissues for pulmonary tissue engineering. Ann Thorac Surg.

[86] Price AP, England KA, Matson AM, Blazar BR, Panoskaltsis-Mortari A (2010). Development of a decellularized lung bioreactor system for bioengineering the lung: the matrix reloaded. Tissue Eng Part A.

[87] Cortiella J, Niles J, Cantu A, Brettler A, Pham A, Vargas G (2010). Influence of acellular natural lung matrix on murine embryonic stem cell differentiation and tissue formation. Tissue Eng Part A.

[88] Jensen T, Roszell B, Zang F, Girard E, Matson A, Thrall R (2012). A rapid lung de-cellularization protocol supports embryonic stem cell differentiation in vitro and following implantation. Tissue Eng Part C Methods.

[89] Wallis JM, Borg ZD, Daly AB, Deng B, Ballif BA, Allen GB (2012). Comparative assessment of detergent-based protocols for mouse lung de-cellularization and recellularization. Tissue Eng Part C Methods.

[90] Daly AB, Wallis JM, Borg ZD, Bonvillain RW, Deng B, Ballif BA (2012). Initial binding and recellularization of decellularized mouse lung scaffolds with bone marrow-derived mesenchymal stromal cells. Tissue Eng Part A.

[91] Ott HC, Clippinger B, Conrad C, Schuetz C, Pomerantseva I, Ikonomou L (2010). Regeneration and orthotopic transplantation of a bioartificial lung. Nat Med.

[92] Akhyari P, Aubin H, Gwanmesia P, Barth M, Hoffmann S, Huelsmann J (2011). The quest for an optimized protocol for whole-heart decellularization: a comparison of three popular and a novel decellularization technique and their diverse effects on crucial extracellular matrix qualities. Tissue Eng Part C.

[93] Witzenburg C, Raghupathy R, Kren SM, Taylor DA, Barocas VH (2012). Mechanical changes in the rat right ventricle with decellularization. J Biomed.

[94] Lu TY, Lin B, Kim J, Sullivan M, Tobita K, Salama G (2013). Repopulation of decellularized mouse heart with human induced pluripotent stem cell-derived cardiovascular progenitor cells. Nat Commun.

[95] Wainwright JM, Czajka CA, Patel UB, Freytes DO, Tobita K, Gilbert TW (2010). Preparation of cardiac extracellular matrix from an intact porcine heart. Tissue Eng Part C.

[96] Remlinger NT, Wearden PD, Gilbert TW. Procedure for decellularization of porcine heart by retrograde coronary perfusion. J Vis Exp. 2012;e50059. 10.3791/50059 PMC3567168 23242494

[97] Momtahan N, Sukavaneshvar S, Roeder BL, Cook AD (2015). Strategies and processes to decellularize and recellularize hearts to generate functional organs and reduce the risk of thrombosis. Tissue Eng Part B.

[98] Methe K, Backdahl H, Johansson BR, Nayakawde N, Dellgren G, Sumitran-Holgersson S (2014). An alternative approach to decellularize whole porcine heart. Biores Open Access.

[99] Jing D, Parikh A, Canty JM, Tzanakakis ES (2008). Stem cells for heart cell therapies. Tissue Eng Part B.

[100] Liu Y, Yang R, He Z, Gao WQ (2013). Generation of functional organs from stem cells. Cell Regen.

[101] Ng SL, Narayanan K, Gao S, Wan AC (2011). Lineage restricted progenitors for the repopulation of decellularized heart. Biomaterials.

[102] Wu SM, Hochedlinger K (2011). Harnessing the potential of induced pluripotent stem cells for regenerative medicine. Nat Cell Biol.

[103] Lenger CJ (2010). iPS cell technology in regenerative medicine. Ann N Y Acad Sci.

[104] van Wijk B, Moorman AF, van den Hoff MJ (2007). Role of bone morphogenetic proteins in cardiac differentiation. Cardiovascular Res.

[105] Perets A, Baruch Y, Weisbuch F, Shoshany G, Neufeld G, Cohen S (2003). Enhancing the vascularization of three-dimensional porous alginate scaffolds by incorporating controlled release basic fibroblast growth factor microspheres. J Biomed Mater Res.

[106] Zisch AH, Lutolf MP, Ehrbar M, Raeber GP, Rizzi SC, Davies N (2003). Cell-demanded release of VEGF from synthetic, biointeractive cell-ingrowth matrices for vascularized tissue growth. FASEB J.

[107] New York Times. Editorial. Discarded kidneys. 2012. http://www.nytimes.com/2012/09/25/opinion/discarded-kidneys.html. Accessed April 2014.

[108] Orlando G, Booth C, Wang Z, Totonelli G, Ross CL, Moran E (2013). Discarded human kidneys as a source of ECM scaffold for kidney regeneration technologies. Biomaterials.

[109] Wagner DE, Bonefant NR, Parsons CS, Sokocevic D, Brooks EM, Borg ZD (2014). Comparative decellularization and recellularization of normal versus emphysematous human lungs. Biomaterials.

[110] Patil PB, Chougule PB, Kumar VK, Almström S, Bäckdahl H, Banerjee D (2013). Recellularization of acellular human small intestine using bone marrow stem cells. Stem Cells Transl Med.

